# Incidence of contralateral cervical metastasis in laryngeal tumors^☆^^☆☆^

**DOI:** 10.1016/j.bjorl.2025.101609

**Published:** 2025-06-04

**Authors:** Arthur Paredes Gatti, Juliana Cristina Pacheco, Nicolas Galat Ahumada, Carlos Neutzling Lehn, Fernando Walder

**Affiliations:** a Universidade Federal de São Paulo (UNIFESP), Departamento de Otorrinolaringologia e Cirurgia de Cabeça e Pescoço, São Paulo, SP, Brazil; b Instituto de Assistência Médica ao Servidor Público Estadual de São Paulo (IAMSPE), Departamento de Cirurgia de Cabeça e Pescoço, São Paulo, SP, Brazil

**Keywords:** Laryngeal neoplasms, Lymphatic metastasis, Neck dissection

## Abstract

**Objective:**

To evaluate if patients with laryngeal SCC homolateral cN+ and contralateral cN− should be submitted to bilateral neck dissection.

**Methods:**

The team reviewed medical records from 135 patients with a diagnosis of laryngeal malignancy between March/2009 and September/2017, analyzing gender, age, tobacco and alcohol comsumption, primary tumor site, neck dissection laterality, clinical and pathological contralaterality, staging, tumor recurrence or late metastasis and survival

**Results:**

We observed that 40.74% were pN+ on at least one side after neck dissection, which 87.27% performed bilateral neck dissection. Of these, 66.67% did not have contralateral metastasis, 87.5% had no previously clinically evident metastasis.

**Conclusion:**

Patients contralateral cN− have a risk <20% for occult metastasis and should not routinely go through bilateral neck dissection.

**Level of evidence:**

Level III.

## Highlights

• The rate of contralateral metastases in supraglottic cancer is low.
• Routine bilateral neck dissection is not recommended when the other site is cN0.
• We still consider bilateral neck dissection only for T4 lesions.

## Introduction

It is well established that the Squamous Cell Carcinoma (SCC) of the larynx and hypopharynx has its spread through the cervical lymphatic system. Over time, there have been changes in the proposals for neck dissection in order to avoiding neoplastic spread beyond its primary site.^1^, ^2^ Currently, neck dissection is indicated when the neck is clinically positive for lymph node metastasis (N+) or when it is negative (N−), but there is a risk greater than 20% for occult metastasis.^3^, ^4^

Neck dissection is not risk free, increasing the morbidity of the procedure: hematoma, seroma, flap necrosis, thromboembolic events, lymphatic fistula, hypoparathyroidism and neurological complications, in addition to increased mortality by 4%. Thus, in addition to the constant improvement in adjuvant methods, it is fundamental to correctly indicate this procedure in order not to add morbidity and mortality risks to the patient.^4^, ^5^

The neck dissection in N+ patients bring increased survival over the morbidity and mortality, regardless of laterality. When the metastatic risk is greater than 20%, the neck dissection of that cervical level is necessary. In the case of clinically evident (cN+) metastasis, unilaterally or bilaterally, comprehensive neck dissection is determinant for the treatment, however there is no consensus about the neck dissection for contralateral clinically negative neck (cN−).^5^, ^6^ Our aim was to evaluate if patients with laryngeal Squamous Cell Carcinoma clinically homolateral N+ and clinically contralateral N− should be submitted to bilateral neck dissection.

## Methods

We reviewed 156 medical records of patients who underwent laryngectomy with a diagnosis of laryngeal malignancy between March/2009 and September/2017 by the Otorhinolaryngology and Head and Neck Surgery Department ‒ Federal University of São Paulo. From this sample, 135 patients were selected (21 excluded due to lack of data, no SCC tumors, hypopharynx cancer or not having undergone neck dissection). The analyzed data segments were gender, age, tobacco and alcohol comsumption, primary tumor site, neck dissection laterality, clinical and pathological contralaterality, staging, tumor recurrence or late metastasis and survival (for this, it was consulted which patients were still alive until this paper and which died due to the disease).

Among the 135 patients, 80 of them was cN− and 55 had clinically positive homolateral neck (chN+). Of these 55 pacients, 48 were submitted to bilateral neck dissection (7 submitted to ipsilateral neck dissection only by unfavorable clinical conditions). Of these, the presence of contralateral metastasis was evaluated in clinically negative cases opposite to the primary tumor.

## Results

There was 115 men (85.19%) and 20 women (14.81%), with a mean age of 61 years. Smoking was present in 132 (97.78%) patients, while alcohol consumption was present in 80 (59.26%). The incidence of tumor recurrence or late metastasis was 15.55% (21 patients).

The prevalent sites of the primary tumor in the casuistic (Table 1) were 51 supraglottic (37.78%); 53 glottic (39.26%); 10 subglottic (7.41%) and 21 transglottic (15.55%). According to the 8th edition of TNM Classification of Malignant Tumours, 10 patients were pT1 (7.41%), 23 pT2 (17.04%), 43 pT3 (31.85%) and 59 pT4 (43.7%) (Table 2). Of these, 55 patients (40.74%) were cN+ on, at least one side in histopathological analysis after neck dissection.

**Table 1:** General primary sites.

Primary sites (General, n = 135)	n (%)
Supraglottic	51 (37.78%)
Glottic	53 (39.26%)
Subglottic	10 (7.41%)
Transglottic	21 (15.55%)

**Table 2:** General pTNM.

TNM (General, n = 135)	n (%)
pT1	10 (7.41%)
pT2	23 (17.04%)
pT3	43 (31.85%)
pT4	59 (43.7%)

In the second analysis, incidence of cN+ in each primary tumor site (Table 3) were 28 supraglottic (50.90%); 11 glottic (20.00%); 8 subglottic (14.55%) and 8 transglottic (14.55%). Also, according to the TNM Classification, 2 patients were pT1 (3.64%), 8 pT2 (14.54%), 17 pT3 (30.91%) and 28 pT4 (50.91%) (Table 4). The incidence of tumor recurrence or late metastasis of these patients was 19.64% (11 patients).

**Table 3:** cN+ primary sites.

cN+ Primary Sites (n = 55)	n (%)
Supraglottic	28 (50.9%)
Glottic	11 (20.0%)
Subglottic	8 (14.55%)
Transglottic	8 (14.55%)

**Table 4:** cN+ pTNM.

cTNM (N+, n = 55)	n (%)
pT1	2 (3.64%)
pT2	8 (14.54%)
pT3	17 (30.91%)
pT4	28 (50.91%)

From this group, 48 (87.27%) received bilateral neck dissection. Of these, 32 (66.67%) did not have contralateral metastasis in the histopathologic report and 28 (87.50%) had no previously clinically evident metastasis, thus showing a <20% risk for contralateral metastasis.

**Fig. 1:**
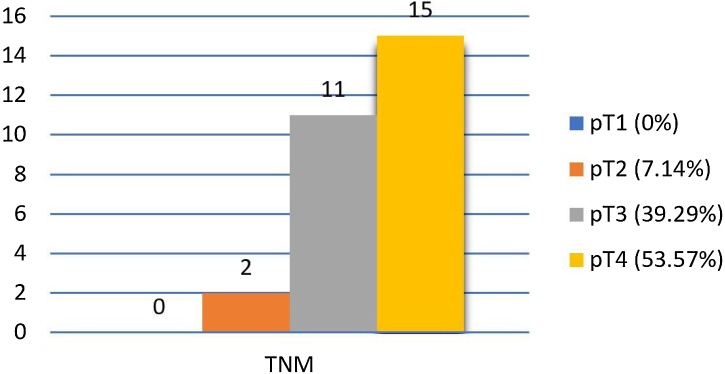
cN− TNM ‒ pathological negative contralateral neck (n = 28).

**Fig. 2:**
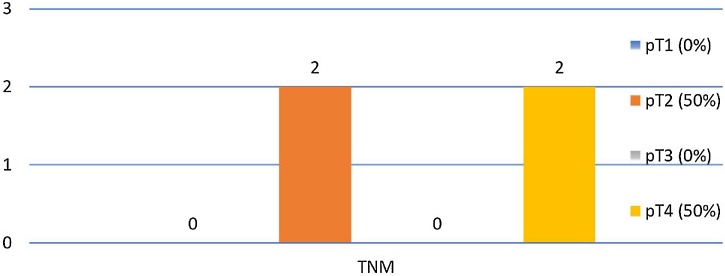
cN− TNM ‒ pathological positive contralateral neck (n = 4).

Of the patients whose sites did not have contralateral metastasis with a cN− (Table 5), 9 were on Supraglottic (32.14%); 9 Glottic (32.14%); 6 Subglottic (21.43%) and 4 Transglottic (14.29%). The TNM Classification (Fig. 1) presented 2 patients as pT2 (7.14%), 11 pT3 (39.29%) and 15 pT4 (53.57%). Of the patients whose sites have contralateral metastasis with a cN− (Table 6), 3 were on Supraglottic (75.00%) and 1 Glottic (25.00%). The TNM Classification (Fig. 2) presented 2 patients as pT2 (50.00%) and 2 pT4 (50.00%).

**Table 5:** cN− primary sites – pathological negative contralateral neck.

cN− Primary Sites – Pathological Negative Contralateral Neck (n = 28)	n (%)
Supraglottic	9 (32.14%)
Glottic	9 (32.14%)
Subglottic	6 (21.43%)
Transglottic	4 (14.29%)

**Table 6:** cN− primary sites – pathological positive contralateral neck.

cN− Primary Sites – Pathological Positive Contralateral Neck (n = 4)	n (%)
Supraglottic	3 (75.0%)
Glottic	1 (25.0%)
Subglottic	0 (0.0%)
Transglottic	0 (0.0%)

**Fig. 3:**
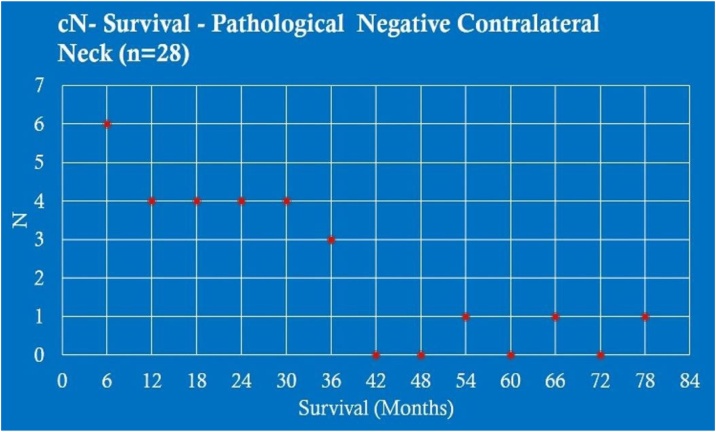
cN− survival ‒ pathological negative contralateral neck (n = 28).

When analyzing the survival in months of patients without contralateral metastasis cN− (28 patients), there were 6 cases with survival between 0–6 months (21.43%); 4 between 6–12-months (14.28%); 4 between 12–18 months (14.28%); 4 between 18–24 months (14.28%); 4 between 24–30 months (14.28%); 3 between 30–36 months (10.71%); 1 between 14–54 months (3.58%); 1 between 60–66 months (3.58%) and 1 between 72–78 months (3.58%) (Fig. 3).

**Fig. 4:**
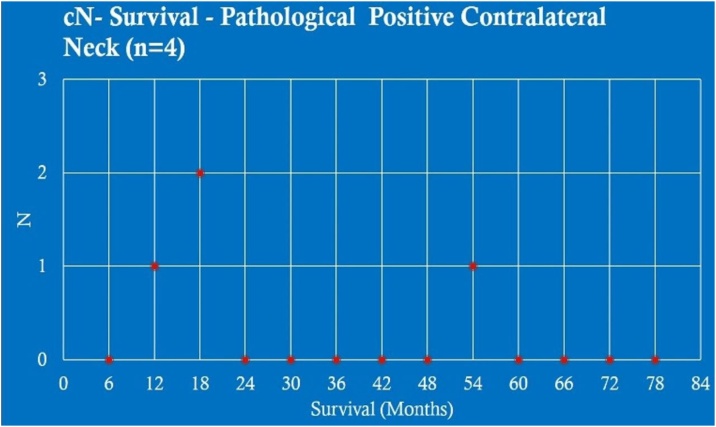
cN− survival ‒ pathological positive contralateral neck (n = 4).

Analyzing the survival in months of patients without contralateral metastasis cN+ (4 patients), there were 1 case with survival between 6–12 months (25.00%); 2 between 12–18 months (50.00%) and 1 between 48–54 months (25.00%) (Fig. 4).

## Discussion

This study’s data shows that 87.5% of cases that the patient had contralateral laryngeal SCC cN− did not really metastasize at this location after histopathological analysis. As the risk was lower than 20%, there would be no indication for routine contralateral neck dissection for these patients.^3^, ^4^

Risk factors (smoking and alcohol consumption), mean age of 60‒65 years and higher prevalence in males were consistent with the latest studies.^2^, ^5^, ^6^, ^7^ Recurrence or late metastasis was mainly due to lost of follow-up by the patient or advanced tumor at the surgical time. A similar incidence was observed between glottic and supraglottic tumors, whereas the literature indicates a prevalence of ⅔ and ⅓, respectively. The low incidence of infraglottic tumors remained consistent with most of the studies analyzed.^8^, ^9^ However, when the pN+ group was evaluated, it was noted the prevalence of supraglottic tumors (50.90%). Regarding tumor staging at the surgical moment, the majority of the cases were at advanced stage, with a predominance of pT4 for both patients in general and in the pN+ groups.

Our department routinely performed bilateral neck dissection regardless of cervical clinical staging before this study (87.27%), finding contralateral metastatic evidence when it was clinically negative only in 12.50% of the cases. Of these patients, 75.00% were supraglottic and specifically of that site, 2 cases were pT2 and 1 was pT4, while the other 25.00% were glottis, with 1 case of pT4.

Overall survival was higher in patients who underwent bilateral neck dissection in the absence of clinically positive contralateral metastasis, however, considering the number of patients being seven times higher than patients who underwent bilateral neck dissection in clinical presence of contralateral metastasis, the relative percentage of patients that still alive in the first group is 37.93%, while the second is 75%.

Despite the statistical findings that showed no obligation to bilateral neck dissection in patients without evident contralateral metastatic, we consider this indication to be significant in cases of advanced tumors (cT4) and when the primary site has crossed lymphatic drainage.

## Conclusion

The judicious performance of neck dissection in laryngeal tumors determine success in cancer treatment of these patients, however the morbimortality of the surgical procedure is the main factor that leads the surgeon to consider which levels should be addressed.^6^, ^9^, ^10^ The literature states the execution of neck dissection on cN+ patients or when the risk of metastasis is greater than 20%.^10^, ^11^ This research demonstrated that there is no such indication for contralateral neck dissection in patients with laryngeal SCC whose contralaterality is cN−.

Without performing contralateral neck dissection in these cases, we are able to avoid the increasing in surgical time, anesthesia exposure, handling of vascular and neurological structures, while also minimizing the incision site with lower exposure to infection, hematoma or seroma as well as reducing the risk of lymphatic fistula.^10^

## Declaration of competing interest

The authors declare no conflicts of interest.
